# Metachromin C, a marine-derived natural compound, shows potential in antitumor activity

**DOI:** 10.7150/ijms.101037

**Published:** 2024-10-07

**Authors:** Pei-Hsuan Chen, Che-Hsin Lee, Chih-Chuang Liaw, Rei-Ting Liang, Mo Aqib Raza Khan, Jen-Ning Tsai, Shin-Yi Huang, Wangta Liu, Wan-Chi Tsai

**Affiliations:** 1 Department of Biological Sciences, National Sun Yat-sen University, Kaohsiung 80424, Taiwan.; 2 Aerosol Science Research Center, National Sun Yat-sen University, Kaohsiung 80424, Taiwan.; 3 College of Semiconductor and Advanced Technology Research, National Sun Yat-sen University, Kaohsiung 80424, Taiwan.; 4 Department of Medical Laboratory Science and Biotechnology, Kaohsiung Medical University, Kaohsiung 80708, Taiwan.; 5 Department of Medical Research, China Medical University Hospital, China Medical University, Taichung 404, Taiwan.; 6 Department of Marine Biotechnology and Resources, National Sun Yat-sen University, Kaohsiung 80424, Taiwan.; 7 Department of Medical Laboratory and Biotechnology, Chung Shan Medical University, Taichung 402, Taiwan.; 8 Clinical Laboratory, Chung Shan Medical University Hospital, Taichung 402, Taiwan.; 9 Department of Biotechnology, Kaohsiung Medical University, Kaohsiung 807, Taiwan.; 10 Department of Laboratory Medicine, Kaohsiung Medical University Hospital, Kaohsiung 807, Taiwan.; 11 Center for Cancer Research, Kaohsiung Medical University, Kaohsiung 807, Taiwan.; 12 Department of Medical Research, Kaohsiung Medical University Hospital, Kaohsiung 807, Taiwan.

**Keywords:** metachromin C, pancreatic cancer, anti-angiogenesis, zebrafish

## Abstract

Metachromin C was first isolated from the marine sponge *Hippospongia metachromia* and has been reported to possess potent cytotoxicity against leukemia cells. However, its antitumor activity and possible mechanisms in pancreatic cancer remain unclear.

The effects of Metachromin C on cell viability were estimated using the 3-(4,5-dimethylthiazol-2-yl)-2,5-diphenyl tetrazolium bromide (MTT) assay. The compound demonstrated a cytotoxic effect on four pancreatic cancer cell lines (PANC-1, BxPC-3, MiaPaCa-2, and AsPC-1). The significant S phase arrest observed with Metachromin C treatment suggests its impact on DNA replication machinery. Metachromin C might interfere with the binding of Topoisomerase I (TOPO I) to DNA, inhibit TOPO I activity, prevent DNA relaxation, cause DNA damage, and consequently activate the DNA repair pathway. Additionally, anti-migration and anti-invasion abilities of Metachromin C were confirmed using the transwell assay. It also inhibited angiogenesis in human endothelial cells by reducing cell proliferation, migration, and disrupting tube formation. Moreover, Metachromin C dose-dependently inhibited the growth of intersegmental vessels, subintestinal vessels, and the caudal vein plexus in a zebrafish embryo model, confirming its inhibitory effect on new vessel formation* in vivo*. Taken together, Metachromin C could not only inhibit the growth of pancreatic cancer cells but also act as an anti-angiogenic compound simultaneously.

## Introduction

Pancreatic cancer, one of the most aggressive cancers, is often referred to as the "king of cancers" due to its difficulty in early detection, lack of effective screening methods, and poor prognosis. Approximately half of the cases are diagnosed at an advanced or metastatic stage 1. Pancreatic ductal adenocarcinoma (PDAC) is the most common type of pancreatic cancer 2. It primarily affects individuals aged 60 years and older, with men being more susceptible. High-risk factors include a family genetic history, chronic pancreatitis, diabetes, obesity, smoking, and alcohol consumption 3.

Currently, chemotherapy remains the mainstay of treatment for pancreatic cancer, with Gemcitabine being the standard first-line treatment for advanced cases. While Gemcitabine is effective in improving survival rates, it also induces resistance, limiting its efficacy 4, 5. In recent years, the combination drug FOLFIRINOX (comprising 5-fluorouracil (5-FU), leucovorin, irinotecan, and oxaliplatin) has become the gold standard for pancreatic cancer treatment, significantly improving overall survival (11.1 months) compared to Gemcitabine (6.8 months) 6, 7. However, the high toxicity of FOLFIRINOX limits its availability to patients, highlighting the urgent need for new treatment options 8. Cell proliferation is crucial for growth, development, and regeneration in eukaryotes and is regulated by "cell cycle checkpoints" to ensure proper cell division. These mechanisms protect DNA replication and mitosis 9. Cancer cells often have defects in genes related to the cell cycle, leading to uncontrolled proliferation and tumor growth. Many antitumor agents block cell cycle progression by causing DNA damage or acting as cell cycle checkpoint inhibitors 10-12. DNA damage responses (DDR) is a group of cellular mechanisms responsible for detecting and repairing DNA damage to maintain genome integrity and stability. In response to DNA damage, various signaling pathways are activated, including DNA repair, DNA damage checkpoints, transcription reactions, and apoptosis 13, 14.

TOPO (Topoisomerases) I activity refers to the enzymatic function of DNA topoisomerase I, an essential enzyme in mammals that plays a critical role in DNA replication, transcription, chromosome segregation, and recombination. 15, 16. They eliminate DNA supercoiling by creating single-strand (type I) or double-strand breaks (type II) 17, 18. Rapidly dividing cancer cells require high levels of TOPO activity, making topoisomerases targets for cancer therapy 19, 20. Camptothecin (CPT), isolated from the Chinese tree *Camptotheca acuminata*, inhibits TOPO I activity but has limited clinical use due to poor water solubility and side effects 21, 22. Derivatives like Topotecan and Irinotecan, approved by the FDA, are used to treat recurrent ovarian cancer and small-cell lung cancer 23. TOPO I inhibitors also play a role in antiangiogenic processes, inhibiting tumor growth by reducing vascular endothelial growth factor (VEGF) production 24-26.

Solid tumors larger than 1-2 mm³ require angiogenesis for growth. Neovascularization supports tumor growth by supplying oxygen and nutrients and providing a pathway for metastasis 27, 28. Anti-angiogenic therapy, such as Bevacizumab, an FDA-approved VEGF antagonist, disrupts the tumor vascular system and is used to treat colorectal and lung cancers 29-35.

Natural products, particularly marine-derived substances, have high biological activity, including antibacterial, anti-inflammatory, and anticancer properties 36-38. Metachromin C, a sesquiterpenoid quinone first isolated from Hippospongia metachromia in 1989, has shown potent effects on leukemia cells but lacks extensive anticancer research 39-41. This study aims to evaluate the anticancer efficacy of Metachromin C in pancreatic cancer and investigate its mechanism of action.

## Materials and Methods

### Cell culture and metachromin C treatment

The four human pancreatic cancer cell lines used in the experiment (PANC-1, BxPC-3, AsPC-1, and MIA PaCa-2) and one human umbilical vein endothelial cell line (HUVEC) were purchased from the Bioresource Collection and Research Center (BCRC, Hsinchu, Taiwan). The immortalized human pancreatic duct epithelial cells (hTERT-HPNE E6/E7) were obtained from the American Type Culture Collection (ATCC, Manassas, VA, USA). PANC-1 and MIA PaCa-2 cells were cultured in Dulbecco's modified Eagle's medium (DMEM) containing 10% fetal bovine serum (FBS) and 1% penicillin/streptomycin (P/S). BxPC-3 and AsPC-1 cells were cultured in RPMI-1640 medium containing 10% FBS, 1% P/S, and 1% sodium pyruvate. HUVEC were cultured in EGM-2 medium (EGM-2 Endothelial Cell Growth Medium-2 Bullet Kit, purchased from Lonza) coated with 0.1% gelatin (ES-006-B, Sigma-Aldrich) and supplemented with 2% FBS and endothelial cell growth supplement (ECGS, CC-4176, Lonza). The hTERT-HPNE E6/E7 cells were cultured in the recommended complete growth medium, which included 5% FBS, 75% DMEM without glucose (D-5030, Sigma-Aldrich), 25% Medium M3 Base (M300F-500, Incell Corp), 10 ng/ml human recombinant EGF, 5.5 mmol/L D-glucose (1 g/L), and 750 ng/ml puromycin. In the experiments, HUVEC from passages 2 to 6 were used. All cells were cultured in a 37°C incubator with 5% CO2. Metachromin C, provided by Professor Chih-Chuang Liaw from the Department of Marine Biotechnology and Resources at National Sun Yat-sen University, has a molecular weight of 358.21, is dissolved in DMSO, and has a stock concentration of 40 mM. Before use, the Metachromin C stock solution was diluted to the desired concentration in the culture medium.

### Cell viability assay

MTT Assay: Cell viability was assessed using the MTT (3-(4,5-dimethylthiazol-2-yl)-2,5-diphenyltetrazolium bromide) assay to evaluate the effects of Metachromin C. PANC-1, BxPC-3, AsPC-1, MIA PaCa-2, and hTERT-HPNE E6/E7 cells were seeded in 96-well plates at a density of 5x10^3^ cells per well and treated with varying concentrations of Metachromin C for 24, 48, and 72 h. Subsequently, the cells were incubated with a 0.5 mg/ml MTT solution (97062-376, VWR life science) at 37°C. After 1 h of incubation, the medium was removed, and 100 μl of DMSO was added to each well to dissolve the formazan crystals. Cell viability was determined by measuring the absorbance at 595 nm using a microplate reader (Molecular Devices).

BrdU Assay: Bromodeoxyuridine (BrdU) is a thymidine analog that is incorporated into newly synthesized DNA during the S-phase of the cell cycle. By using an anti-BrdU antibody, proliferating cells can be detected through an ELISA-based assay. The HUVEC cells were seeded at a density of 2 × 10^4^ cells per well in 96-well plates and treated with the specified concentrations of Metachromin C for 24 h. Following the instructions provided by the BrdU Cell Proliferation Assay Kit (2752, Sigma-Aldrich), absorbance was measured at 450 nm using a microplate reader (Molecular Devices).

Click-iT EdU Assay: The EdU assay was conducted using the Click-iT EdU imaging kit (C10339, Invitrogen) with Alexa Fluor 594 Azides (red fluorescence). According to the manufacturer's instructions, HUVEC cells were seeded at a density of 5 × 10^4^ cells per well on round coverslips in 24-well plates and treated with the specified concentrations of Metachromin C for 24 h. Two hours before the end of the treatment, half of the medium was replaced with an EdU labeling medium. After incubating the plates at 37°C for 2 h, cells were fixed and permeabilized. The medium was removed, and 1 ml of PBS containing 3.7% paraformaldehyde (30525-89-4, Sigma-Aldrich) was added, incubating at room temperature for 15 min. The fixative was then removed, and cells were washed with 1 ml of PBS containing 3% BSA. After removing the wash solution, 1 ml of PBS containing 0.5% Triton X-100 was added, and cells were incubated at room temperature for 20 min. The permeabilization buffer was removed, and cells were washed twice with 1 ml of PBS containing 3% BSA. Finally, 0.5 ml of Click-iT reaction cocktail was added, ensuring it covered the coverslip evenly, and incubated in the dark for 30 min. The reaction cocktail was then removed, and cells were washed once with PBS containing 3% BSA. For mounting, 10 μl of mounting medium with DAPI was placed on a microscope slide, and the coverslip with cells was placed on the mounting medium. The cells were then observed under a fluorescence microscope.

### Cell cycle analysis

Thymidine was used to synchronize cells in the G1 phase. Briefly, PANC-1 and BxPC-3 cells were seeded at a density of 5 × 10^5^ cells in 6 cm culture dishes. They were then treated with varying concentrations of Metachromin C for 48 h. The cells were harvested using 0.05% Trypsin-EDTA and centrifuged at 1300 rpm for 10 min to remove the supernatant. The cells were washed with PBS and resuspended. They were then fixed in 70% ethanol overnight. The next day, the fixed cells were washed with PBS and incubated in a solution containing 50 µg/ml RNase A, 40 µg/ml propidium iodide (PI, 40017, Biotium), and PBS at 37°C with gentle shaking at 50 rpm for 1 h in the dark. Finally, cell cycle distribution was analyzed using a BECKMAN COULTER Cytomics™ FC500 Flow Cytometer and CXP analysis software.

### DNA binding assay

Calf thymus DNA absorption titration study: To visualize the binding mode between CT DNA and the target compound Metachromin C, absorption studies were carried out. In the current study, we predict the binding fashion as to whether CT DNA binds with the compound in an intercalative or groove manner. A stock solution of CT DNA (3 mM) was prepared in a 10 mM Tris-HCl buffer solution at a pH of 7.2 since a more acidic pH environment is likely to denature the CT DNA strands. A UV analysis for free CT DNA was done before the experiment which showed two bands at 260 and 280 nm (A260 and A280) and their ratio corresponded to 1:9:1 which indicated that the CT DNA was sufficiently free of protein. The title compound was taken in a mixture of 5 % DMSO and 95 % Tris-HCl buffer medium and the titration was done by fixing the concentration of the compound (2.0 µM) while adding CT DNA in an incremental manner (5- 30 µM). Emission Spectral Studies: A competitive binding with Ethidium Bromide (EB). To further understand the binding nature of CT DNA to Metachromin C, emission spectral studies were performed. The CT DNA and EB were pretreated 2 h before the start of the experiment. The emission wavelength was fixed at 510 nm and the fluorescence intensity pattern was seen at 615 nm.

### Topo I mediated DNA relaxation assay

The assay was performed using a TopoGen human topoisomerase I assay kit (TG1015-1A, TopoGEN). Following the manufacturer's protocol, a standard relaxation reaction mixture (20 μl) was prepared containing Topo I reaction buffer (10 mM Tris-HCl pH 7.9, 1 mM EDTA, 0.15 M NaCl, 0.1% BSA, 0.1 mM Spermidine, 5% glycerol), 1μl of pHOT1 (supercoiled plasmid DNA), 1 unit of human Topo1 (Topogen, USA) and indicated concentrations of Metachromin C were incubated at 37°C for 30 mins. Reactions were terminated by adding 4 μl of 5X stop buffer (0.125% bromophenol blue, 25% glycerol, 5% Sarkosyl). The relaxed DNA products were analyzed via electrophoreses through 1% agarose gel in 1X TAE buffer at 2.5V/cm for 2 h. The relaxed pHOT1 (marker DNA) was loaded as a relaxed DNA reference. The gel was stained with ethidium bromide and photographed using the Luminescence image system.

### Single-cell gel electrophoresis (SCGE)

The SCGE, also known as the comet assay, is used to detect DNA damage at the level of individual cells. PANC-1 and BxPC-3 cells were seeded at a density of 1.5 × 10^5^ cells in 6-well plates and treated with the specified concentrations of Metachromin C. For the alkaline electrophoresis positive control group, cells were treated with 100 μM Camptothecin (S1288, Selleck Chemicals) for 24 h, and for the neutral electrophoresis positive control group, cells were treated with 200 μM Hydrogen peroxide (H_2_O_2_, 31642, Honeywell) at 4°C for 30 min. Cells were then collected, washed, and centrifuged. The supernatant was removed, and the cells were resuspended in Dulbecco's phosphate-buffered saline (DPBS, 3-05K29-I, BioConcept). A 1% normal melting point agarose (NMP) solution was prepared and heated in a microwave for 2 min. An 85 μl aliquot of the 1% NMP was placed on a slide, covered with a coverslip to form a transparent gel, and allowed to dry. After drying, the coverslip was removed. A 0.5% low melting point agarose (LMP) solution was prepared and heated in a microwave for 2 min. A 10 μl aliquot of the cell suspension was mixed with 75 μl of 0.5% LMP and placed on the slide containing the 1% NMP. A coverslip was placed over the mixture, and once dried, the coverslip was removed. The slides were placed flat in freshly prepared lysis solution (2.5 M NaCl, 100 mM EDTA-2Na, 10 mM Tris, and 1% Triton X-100, pH 10) at 4°C for 1 h to remove cell membranes and proteins. The slides were gently removed from the lysis solution and placed in an electrophoresis tank containing alkaline (200 mM NaOH and 1 mM EDTA, pH > 13) or neutral (89 mM Tris, 89 mM boric acid, and 2 mM EDTA, pH 7) electrophoresis buffer at 4°C for 10 min to allow DNA unwinding. The electrophoresis tank was then moved to an ice box and electrophoresis was performed using alkaline (30V, 15 min) or neutral (21V, 45 min) conditions. After electrophoresis, the slides were immersed in neutralization buffer at 4°C for 5 min, then dehydrated in methanol for 5 min and air-dried. The slides were stained with 40 μl of the nucleic acid stain PI and observed under a fluorescence microscope. Images were analyzed using ImageJ with the OpenComet plugin, quantifying the tail length of 50 cells per group.

### Immunofluorescence (IF)

PANC-1 and BxPC-3 cells were seeded at a density of 5×10^4^ cells per well on round coverslips in 24-well plates and treated with the specified concentrations of Metachromin C for 48 h. After removing the medium, the cells were fixed with 3.7% paraformaldehyde, washed twice with 3% BSA, and permeabilized with 0.5% Triton X-100. The cells were then washed twice with 3% BSA. Primary antibodies prepared in 3% BSA were added and incubated overnight at 4°C in the dark. The following day, the cells were washed with PBS, and the corresponding secondary antibodies prepared in 3% BSA were added and incubated at room temperature for 2 h. The cells were then washed for 5 min with PBS, mounted with a mounting medium containing DAPI (P36931, Invitrogen), and kept at 4°C in the dark. The slides were observed the next day using a fluorescence microscope.

### Western blotting

Cells were lysed in RIPA lysis buffer containing protease and phosphatase inhibitors. The cell lysates were centrifuged at 13,000 rpm for 30 min at 4°C, and the supernatant was collected as the total cell lysate. Total protein concentration was quantified using the Bio-Rad Protein Assay Kit. Depending on the molecular weight of the target proteins, proteins were separated by SDS-PAGE using an appropriate gel concentration and then transferred to a polyvinylidene difluoride (PVDF) membrane. The membrane was blocked with 5% non-fat milk or 5% BSA for 1 h. Target proteins were detected by incubating the membrane overnight at 4°C with the following primary antibodies: anti-p-ATM, anti-p-ATR, anti-p-p53, anti-p-BRCA1, anti-p-chk1, anti-p-chk2, and anti-p-Histone H2A.X (all at 1:1000 dilution, from Cell Singling Technology). Anti-β-actin (1:5000, from Santa Cruz Biotechnology, Santa Cruz, CA, USA) was used as a loading control. The membrane was then washed with Tris-buffered saline containing Tween 20 (TBST) and incubated for 1 h at room temperature with horseradish peroxidase-conjugated secondary antibodies (Santa Cruz Biotechnology) against the primary antibodies. Protein expression was detected using an enhanced chemiluminescent (ECL, PI34096, Thermo Scientific) reagent substrate and visualized with a chemiluminescence/fluorescence imaging system.

### Transwell migration and invasion assay

In the migration assay, 2 × 10^4^ cells/well were seeded into the upper chamber of Transwell inserts (8.0 µm, 353097, Corning), with different concentrations of Metachromin C and DMSO as the vehicle control prepared in a serum-free medium. The lower chamber was filled with medium containing 10% FBS, serving as a chemoattractant. After an 8 h incubation, cells that had migrated through the membrane were stained and counted. The Transwell inserts were removed, the medium in the wells was discarded, and the cells were washed twice with PBS. The cells were then fixed and permeabilized with 10% Triton X-100 and 4% paraformaldehyde for 30 min, followed by staining with 0.1% crystal violet for 30 min. The non-migrated cells on the upper surface were gently wiped off with a cotton swab, and the migrated cells were counted under a microscope. For the invasion assay, the upper chamber of the Transwell was coated with Matrigel to mimic the *in vitro* environment of invasive cancer cells degrading and penetrating the extracellular matrix (ECM) barrier. The Matrigel stock solution (22 mg/ml, 354262, Corning) was diluted to a 300 μg/ml coating solution in a serum-free medium. Using pre-cooled pipettes and tips, 100 μl of the diluted Matrigel coating solution was carefully added to the Transwell inserts. The plates with Matrigel-coated inserts were incubated at 37°C for 2 h. Then, 2 × 10^4^ cells/well were seeded into the upper chamber of the Matrigel-coated Transwell inserts, with different concentrations of Metachromin C and DMSO as the vehicle control prepared in a serum-free medium. The lower chamber was filled with medium containing 10% FBS. After a 24-hour incubation, cells that had invaded through the Matrigel were stained and counted.

### Tube formation assay

Growth Factor Reduced (GFR) Matrigel (354230, Corning) were added to a 96-well plate, ensuring the Matrigel was evenly distributed in the wells, and incubated at 37°C for 30 min to allow the Matrigel to solidify. Different concentrations of Metachromin C and DMSO as a control group were prepared in 100 μl of medium. A mixture of 1.5×10^4^ cells/well and the drugs were then seeded onto the Matrigel-coated 96-well plate and incubated at 37°C for 24 h. The results were immediately observed under a microscope, followed by fixation and staining. Cells were fixed with 100 μl of 4% paraformaldehyde at room temperature for 15 min, the fixative was removed, and cells were washed twice with DPBS. Subsequently, cells were stained with 100 μl of 0.1% crystal violet at room temperature for 30 min, washed twice with DPBS, and observed under a microscope. HUVEC cells were seeded onto a Matrigel-coated 96-well plate and incubated at 37°C for 8 h to allow tube formation, simulating pre-existing vessels in the human body. The formed tubes were then treated with different concentrations of Metachromin C and DMSO as a control group, incubated at 37°C for 6 h, and observed under a microscope. Vascular images were analyzed using Wimasis software (Onimagin Technologies SCA, Cordoba, Spain).

### Zebrafish maintenance

The transgenic zebrafish Tg (fli1: EGFP) fish line was obtained from the Taiwan Zebrafish Core Facility at the National Health Research Institute and maintained at the Zebrafish Core Facility at Kaohsiung Medical University. The fishline was Artemia and maintained on a 14 h light/10 h dark cycle at 28.5 °C. The embryos were collected by mating the zebrafish. The collected embryos were maintained in egg water in an incubator at 28.5 °C. All zebrafish experiments were performed in an association for Assessment and Accreditation of Laboratory Animal Care International (AAALAC)-accredited Zebrafish Core Facility, Kaohsiung Medical University, Kaohsiung, Taiwan.

### Zebrafish angiogenesis assay

At 15 h post-fertilization (hpf), zebrafish embryos were treated with the specified concentration of Metachromin C and simultaneously administered N-phenylthiourea (PTU) to inhibit melanin pigmentation in the embryos. At 30 hpf, Pronase (9036-06-6, Roche) was used for 5 min to soften the chorion until it detached. The embryos were then immobilized in 3% methylcellulose on concave slides, and a drop of 0.01% Tricaine (A5040, Sigma-Aldrich) anesthetic was added to anesthetize them for 3-5 min. The embryos were then transferred to a fluorescence microscope for observation and imaging. After imaging, the embryos were placed back into a 24-well plate and observed again at 3 days post-fertilization (dpf). Imaging was performed using a modular stereo microscope for fluorescence imaging (MZ10F, Leica, Singapore) equipped with a LEICA 1.0x lens (10445930, Leica, Singapore) and Metavue software (version 7.8.0.0).

### Statistical analysis

In this study, experimental data were presented as Mean ± standard deviation (SD), except for the Comet assay, which is presented as Mean ± mean of standard error (SEM). A paired Student's t-test was used to calculate whether the differences between the control and experimental groups were statistically significant. The levels of significance were indicated as follows: * p < 0.05, ** p < 0.01, *** p < 0.001.

## Results

### Metachromin C causes stagnation and subsequent inhibition of growth in pancreatic cancer cells in the S phase

**Figure 1:**
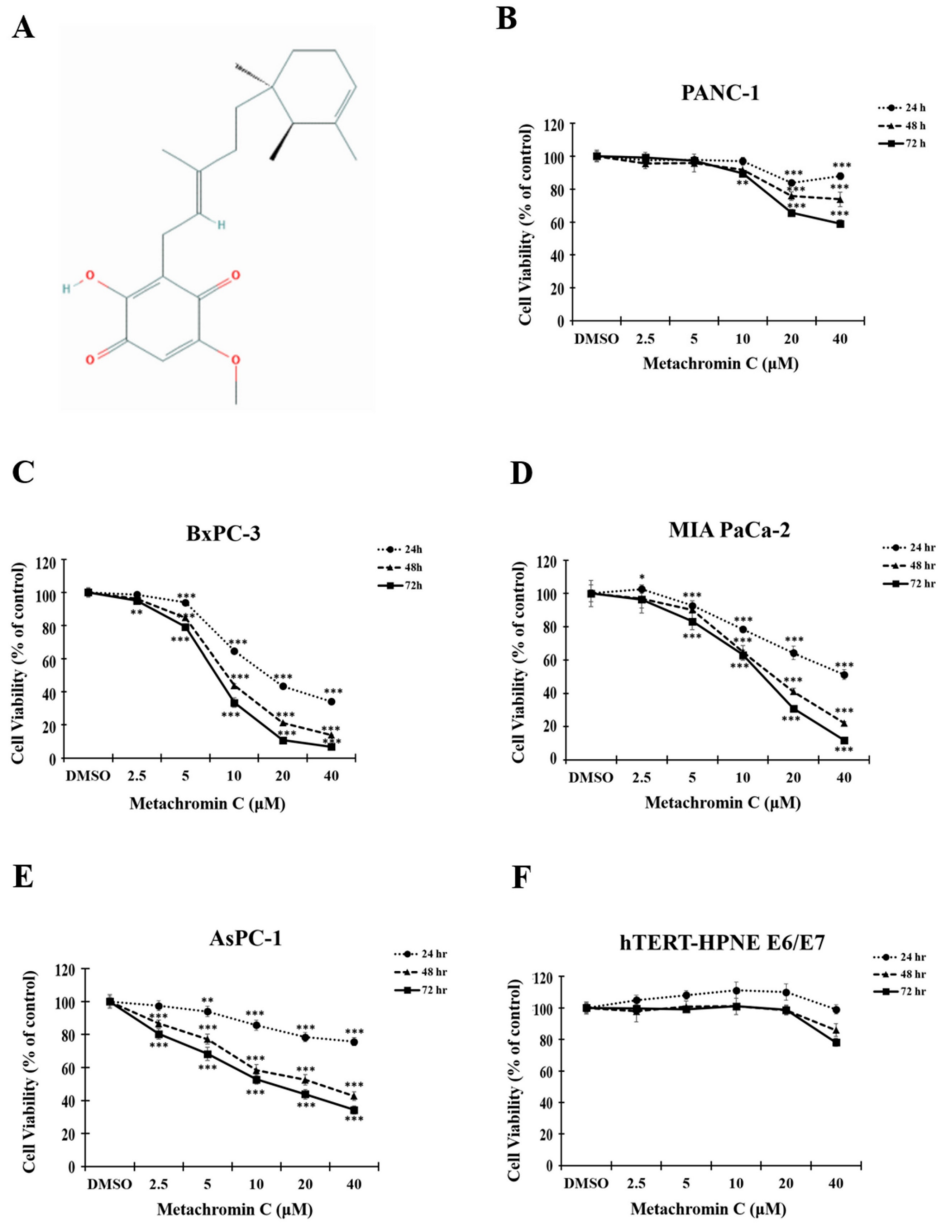
** Metachromin C inhibits cell viability in pancreatic cancer cells while having a limited effect on normal pancreatic cells.** (A) Chemical structure of Metachromin C. MTT assay demonstrated that Metachromin C inhibits the viability of (B) pancreatic cancer PANC-1, (C) BxPC-3, (D) MIA PaCa-2, (E) AsPC-1 cells, and (F) normal human pancreatic duct epithelial hTERT-HPNE E6/E7 cells in a dose- and time-dependent manner. Data were shown with mean ± standard deviation (SD) (n=6). * p < 0.05, ** p < 0.01, *** p < 0.001.

**Figure 2:**
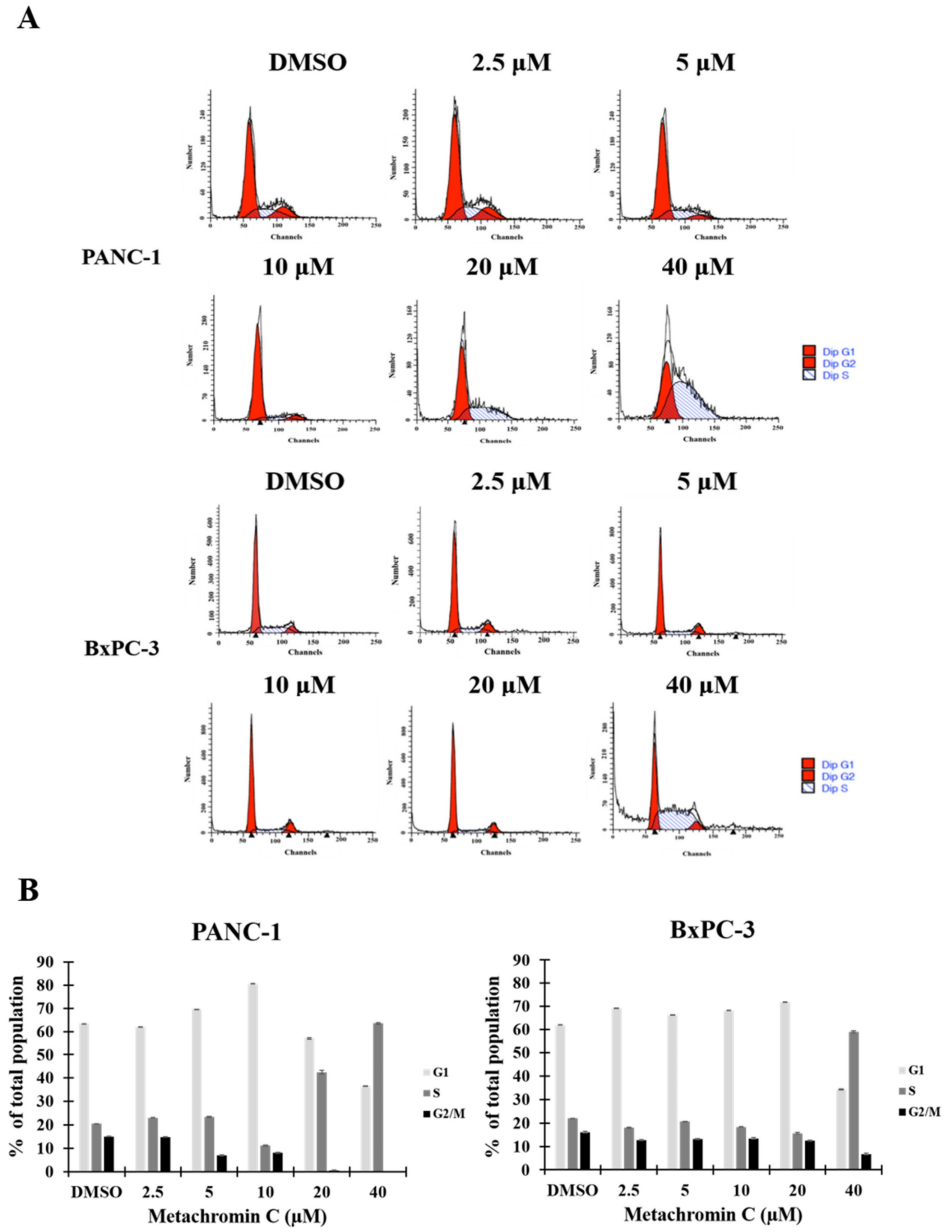
** Effects of Metachromin C on cell cycle distribution in pancreatic cancer cells.** (A) PANC-1 and BxPC-3 cells were treated with 2.5, 5, 10, 20, and 40 µM Metachromin C for 48 h, stained with propidium iodide, and analyzed for cell cycle distribution by flow cytometry. (B) Data quantification plots represent the percentage of cells in the G1, S, and G2/M phases of the cell cycle. Data were shown with mean ± SD (n=3). * p < 0.05, ** p < 0.01, *** p < 0.001.

The chemical structure of Metachromin C is shown in Figure 1 A. The effect of Metachromin C on cell viability in PDAC cell lines (PANC-1, BxPC-3, MIA PaCa-2, and AsPC-1) was determined using the MTT assay. Metachromin C was tested at concentrations of 2.5, 5, 10, 20, and 40 μM, with DMSO as the experimental control group, over treatment periods of 24, 48, and 72 hours. The results showed that Metachromin C significantly inhibited cell viability in a dose- and time-dependent manner (Fig. 1B, C, D, and E). The IC50 values for Metachromin C were 16.9 μM, 9.2 μM, and 8.2 μM for BxPC-3 cells; 16.2 μM and 14.1 μM for MiaPaCa-2 cells; and 24.5 μM and 13.3 μM for AsPC-1 cells. To confirm whether Metachromin C is also cytotoxic to normal pancreatic cells (Fig. 1 F), MTT assays were performed using the normal pancreatic ductal cell lines hTERT-HPNE E6/E7. Metachromin C only slightly inhibits cell activity in normal pancreatic cells. Gemcitabine-sensitive BxPC-3 and resistant PANC-1 cell lines were selected as cell models for subsequent experiments. To further investigate the possible mechanism of Metachromin C in pancreatic cancer cell growth, we analyzed its effect on PANC-1 and BxPC-3 cell cycle progression (Fig. 2 A). The two cell lines were synchronized in the G1 phase by bithymidine blockade, treated with Metachromin C for 48 hours, and the distribution of cell cycle stages was analyzed by flow cytometry. The results showed a significant increase in the proportion of PANC-1 and BxPC-3 cells in the S phase after treatment with Metachromin C compared to the control group (Fig. 2 B). These results suggest that Metachromin C inhibits cell cycle progression by blocking pancreatic cancer cells in the S phase, resulting in inhibited cell growth.

### Metachromin C inhibits topoisomerase I activity and induces DNA single-strand breaks

**Figure 3:**
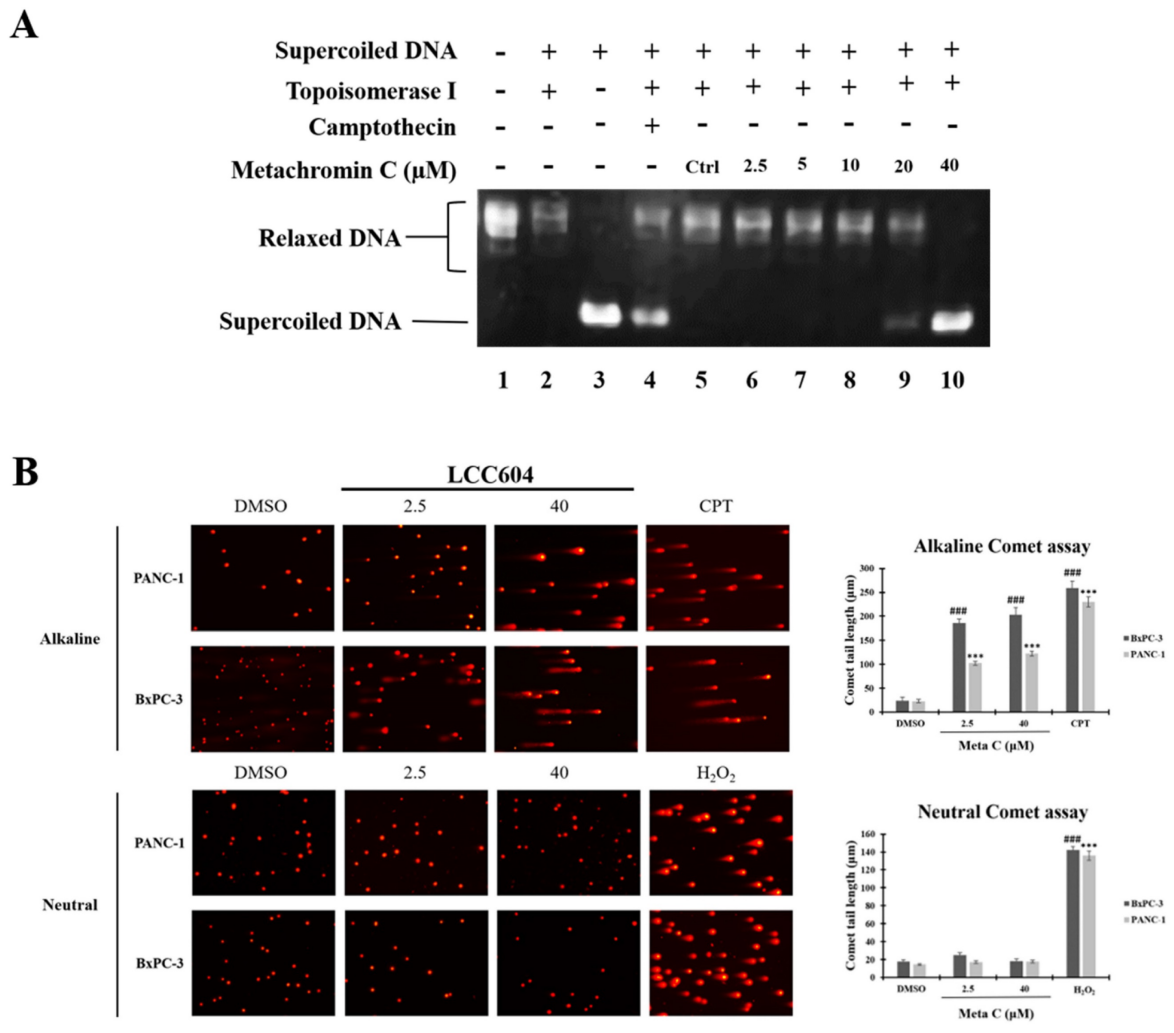
** Metachromin C inhibited topoisomerase I activity and induced DNA single-strand breaks (SSBs).** Detection of topo I-mediated supercoiled pHOT1 DNA relaxation in a cell-free system. Lane 1, Relaxed DNA marker; Lane 2, Supercoiled pHOT-1 DNA was incubated with topoisomerase I; Lane 3: Supercoiled pHOT1 DNA alone; Lane 4, Supercoiled pHOT-1 DNA was incubated with topoisomerase I combined 100 µM Camptothecin (CPT, topoisomerase I inhibitor as positive control); Lane 5, Supercoiled pHOT-1 DNA was incubated with topoisomerase I combined DMSO (solvent control); Lane 6-10, Supercoiled pHOT-1 DNA was incubated with topoisomerase I combined 2.5, 5, 10, 20 and 40 µM Metachromin C. (B) PANC-1 and BxPC-3 cells were exposed to Metachromin C (2.5 and 40 μM) for 24 h, and DNA damage was evaluated with alkaline and neutral comet assays. DMSO was used as the negative control, and 100 μM CPT and 200 μM hydrogen peroxide (H_2_O_2_) were used as positive controls in alkaline and neutral electrophoresis, respectively. Comet tail length was calculated to assess DNA damage. Data were shown with mean ± standard error of the mean (SEM) (n=50). PANC-1, * p < 0.05, ** p < 0.01, *** p < 0.001; BxPC-3, # p < 0.05, ## p < 0.01, ### p < 0.001.

The S phase of the cell cycle is when DNA replication occurs. Metachromin C induces pancreatic cancer cells to arrest in the S phase, prompting investigation into its effect on DNA replication mechanisms. Topoisomerases play an essential role in DNA replication. We performed a molecular docking strategy and we identified the potential binding site of Metachromin C with TOPO 1, showing that it might compete with the DNA binding site of TOPO1 (Fig. S1). Determine whether Metachromin C can bind to DNA by using a DNA binding assay (Fig. S2). The results show that Metachromin C can bind to DNA via intercalation mode. To confirm whether Metachromin C can inhibit TOPO I activity, we performed a Topo I assay in a cell-free system (Fig. 3). Purified human topoisomerase I enzyme was used to examine the relaxation of supercoiled plasmid DNA (pHOT1), with camptothecin (CPT) as a positive control. The results showed that Metachromin C inhibited Topo I enzyme activity, preventing the conversion of supercoiled DNA to a relaxed form (lanes 9-10) (Fig. 3A). TOPO I causes single-strand DNA (ssDNA) breaks, while TOPO II causes double-strand DNA (dsDNA) breaks. The comet assay was used to detect ssDNA and dsDNA breaks. Metachromin C at concentrations of 2.5 and 40 μM, with DMSO as the control, was tested for 24 hours using alkaline and neutral comet assays (Fig. 3B). DNA tailing was observed in the alkaline comet assay but not in the neutral comet assay, suggesting that Metachromin C causes single-strand DNA breaks. These results suggest that Metachromin C inhibits TOPO I function, leading to DNA single-strand fragmentation.

### Long-term treatment of metachromin C transforms single-strand breaks into toxic double-strand breaks and activates DNA repair mechanisms

**Figure 4:**
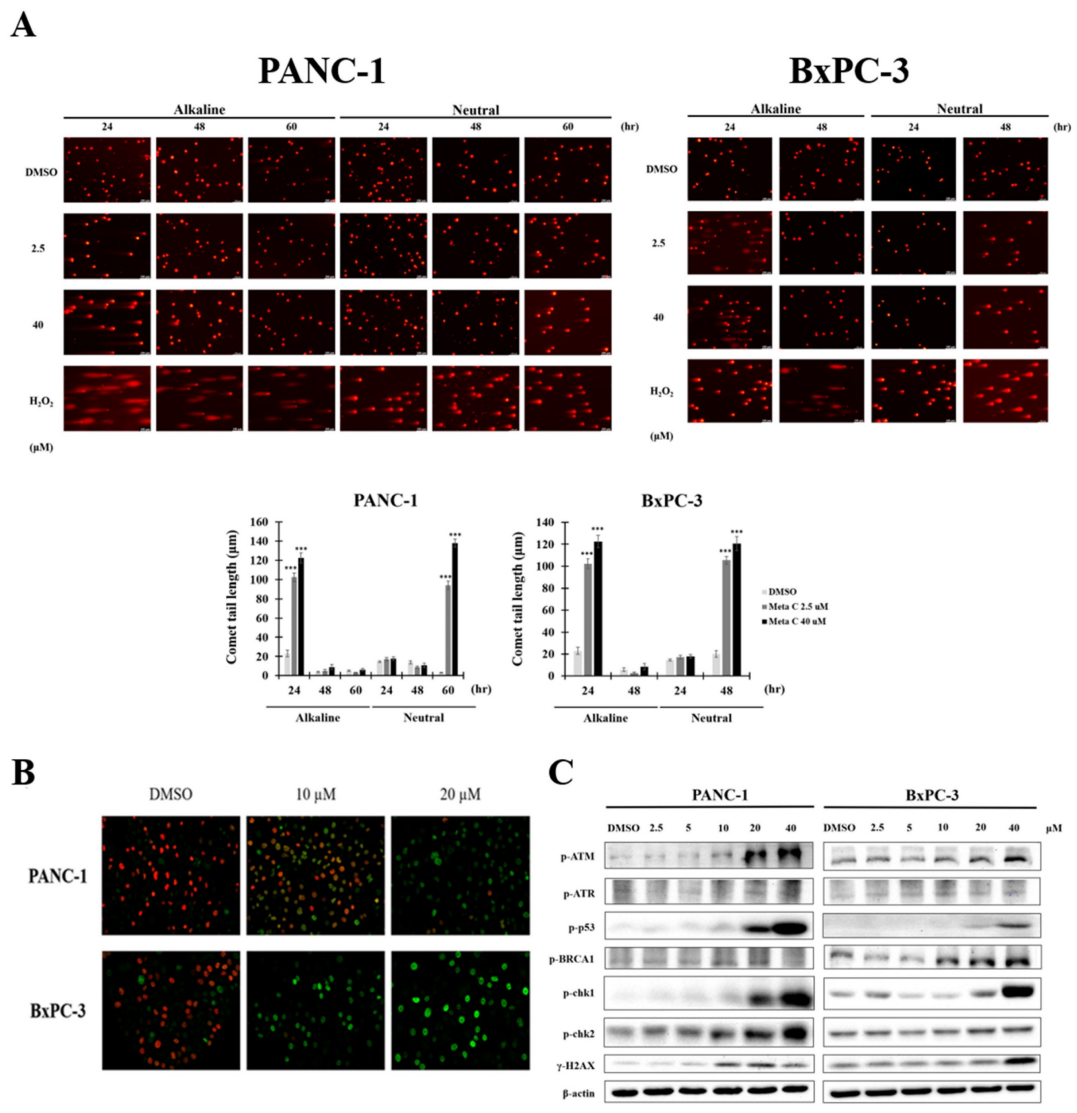
** Prolonged treatment of Metachromin C made SSBs convert to toxic double-strand breaks (DSBs) and activated DNA repair machinery.** PANC-1 cells were exposed to Metachromin C (2.5 and 40 μM) for 24, 48, and 60 h, and DNA damage was evaluated with alkaline and neutral comet assays. BxPC-3 cells were exposed to Metachromin C (2.5 and 40 μM) for 24 and 48 h, and DNA damage was evaluated with alkaline and neutral comet assays. DMSO was used as the negative control and 200 μM H_2_O_2_ was used as the positive control in alkaline and neutral electrophoresis, respectively. Comet tail length was calculated to assess DNA damage. Data were shown with mean ± SEM (n=50). * p < 0.05, ** p < 0.01, *** p < 0.001. (B) PANC-1 and BxPC-3 cells were co-stained with γH2AX and EdU after Metachromin C treatment for 48 h and observed in a confocal microscope. The merged picture showed the overlapping of γH2AX and EdU in PANC-1 indicating DNA damage occurred while cells were undergoing DNA replication. (C) Western blots of indicated proteins were performed on whole-cell extracts from PANC-1 and BxPC-3 cells 48 h after treatment with Metachromin C (2.5, 5, 10, 20, and 40 µM ). Actin provides the loading control.

Unrepaired single-strand breaks (SSBs) can lead to double-strand breaks (DSBs) when cells undergo DNA replication. To evaluate whether long-term treatment with Metachromin C causes more lethal damage, we conducted comet assays with PANC-1 and BxPC-3 cells treated for 24, 48, and 60 hours. In PANC-1 cells, double-strand breaks were observed at 60 hours of treatment (Fig. 4 A), while in BxPC-3 cells, double-strand breaks were observed at 48 hours (Fig. 4 A). The presence of γH2AX, a DNA damage marker, was significantly induced after 48 hours of Metachromin C treatment in PANC-1 and BxPC-3 cells, indicating DNA damage (Fig. 4 B). Western blot analysis showed that Metachromin C induced the expression of DNA repair proteins (ATM, ATR, p53, BRCA1, ChK, ChK2) after 48 hours of treatment (Fig. 4 C). These results suggest that prolonged Metachromin C treatment leads to severe double-strand breaks and activates DNA repair pathways.

### Metachromin C inhibits the proliferation, migration, and tube-forming ability of HUVECs without affecting pre-existing blood vessels

Tumor endothelial cells exhibit high TOPO I activity, and Metachromin C, as a TOPO I inhibitor, was tested for its effect on endothelial cells. The BrdU and EdU assays showed that Metachromin C reduced HUVEC cell proliferation in a dose-dependent manner (Fig. 6 A, and B). Transwell migration assays indicated that Metachromin C decreased HUVEC migration ability (Fig. 6 C). *In vitro* tube formation assays showed that Metachromin C inhibited the formation of tubular structures at higher doses (10 μM) (Fig. 6 D). However, Metachromin C did not affect pre-existing tubular structures, suggesting its selective effect on newly forming blood vessels (Fig. 6 E). These results indicate that Metachromin C exhibits anti-angiogenic properties by hindering HUVEC proliferation, migration, and tube formation.

### Anti-angiogenic effect of metachromin C on genetically transgenic zebrafish* in vivo*

The anti-angiogenic potential of Metachromin C was tested using transgenic zebrafish Tg (flil1a: EGFP) (Fig. 6 A). Survival analyses showed that Metachromin C was non-toxic at concentrations of 1 and 2 μM (Table 1). Treatment of 15 hours post-fertilization (hpf) zebrafish embryos with Metachromin C was found to reduce the number of complete intersegmental vessel (ISV) connections to the dorsal longitudinal anastomotic vessel (DLAV) while increasing the number of defective ISV connections to the myoseptum (Fig. 6 B). Meanwhile, Metachromin C reduced the area of the caudal vein plexus (CVP) and inhibited CVP formation (Fig. 6 C). Following culture to 3 days post-fertilization (dpf), Metachromin C was observed to decrease the number of vascular intersection points and the length of the subintestinal vessels (SIVs), as well as diminish the growth of SIV branch vessels (Fig. 6 D). These results suggest that Metachromin C inhibits angiogenesis in vivo in a zebrafish model.

### Metachromin C exerts anti-metastatic potential by inhibiting the migration and invasion ability of pancreatic cancer cells

**Figure 5:**
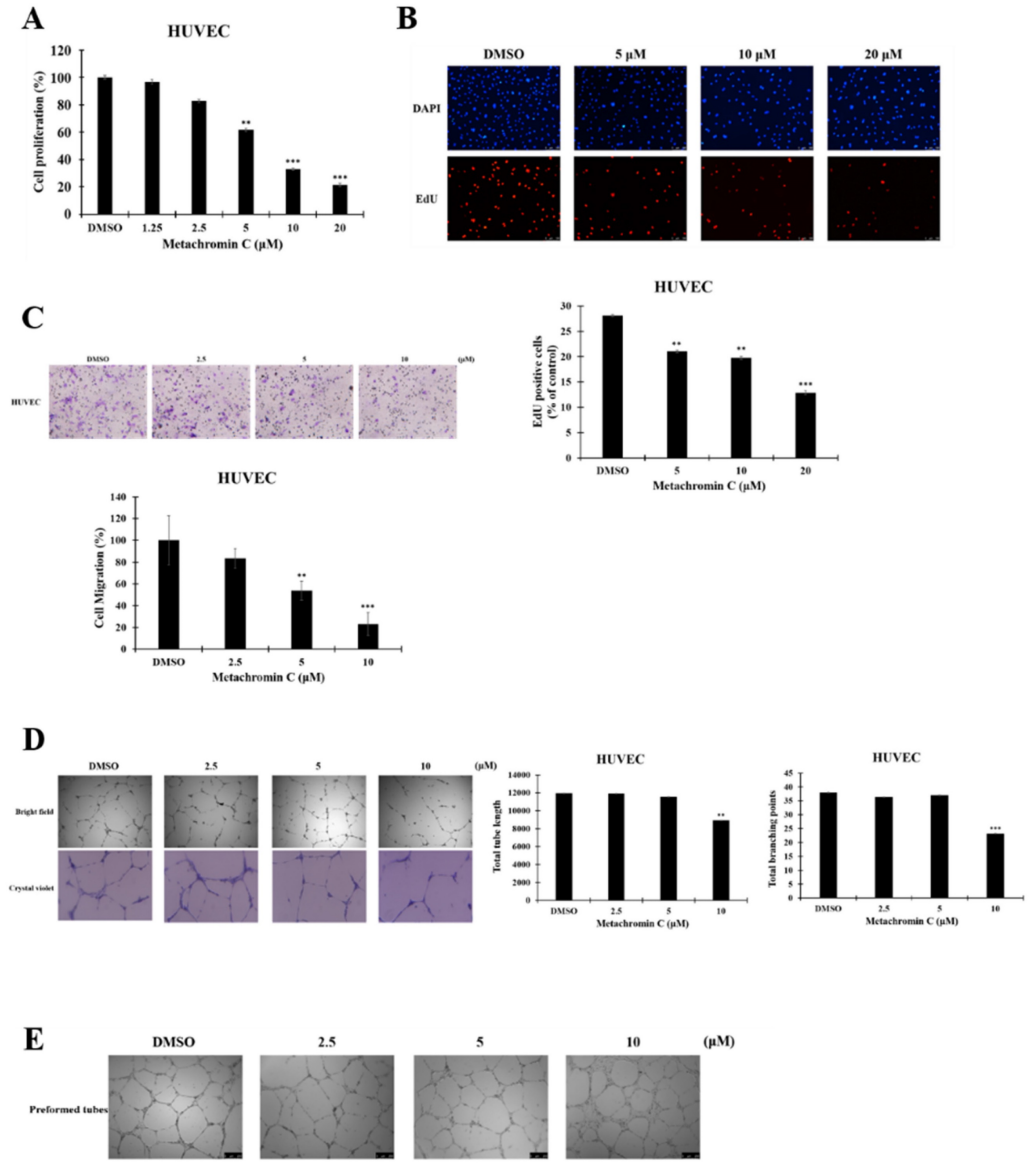
** Metachromin C exhibits anti-proliferative effects on HUVEC, impeding their migration and tube-forming capabilities, while having no impact on pre-existing blood vessels formed by HUVEC.** BrdU incorporation measured by ELISA in HUVEC following 24 h treatment with the indicated dose of Metachromin C. (B) The EdU stain assay was employed to detect EdU-positive cells embedded in red fluorescent protein. The count of red fluorescent EdU-positive cells was observed using an inverted fluorescence microscope. DAPI-stained cell nuclei (in blue) were utilized as a background stain to determine the percentage of EdU-positive cells and node cells. (C) Transwell-migration assay of HUVEC was conducted to investigate cell migration after indicated concentrations of Metachromin C treatment for 8 h. The migrated cells were stained with crystal violet and counted by ImageJ. Cell migration rate was normalized with the DMSO control group. (D) Different concentrations of Metachromin C were mixed with HUVEC cells and then inoculated onto matrigel for a 24 h incubation period. The tubular structures formed by HUVEC were observed under an inverted microscope (40× magnification; scale bar = 250 μm), and the total tube length was quantified using Wimasis software, along with the measurement of the number of branch points. Additionally, the tubular structures of HUVEC were visualized under an inverted microscope (100× magnification; scale bar = 250 μm) following crystal violet staining. (E) Different concentrations of Metachromin C were added after tubes were established on matrigel for 8 h, and incubated for another 6 h. Tubular structures were observed by inverted microscope (40× magnification; scale bar = 250 μm). Data were shown with mean ± SD (n=3). * p < 0.05, ** p < 0.01, *** p < 0.001.

**Figure 6:**
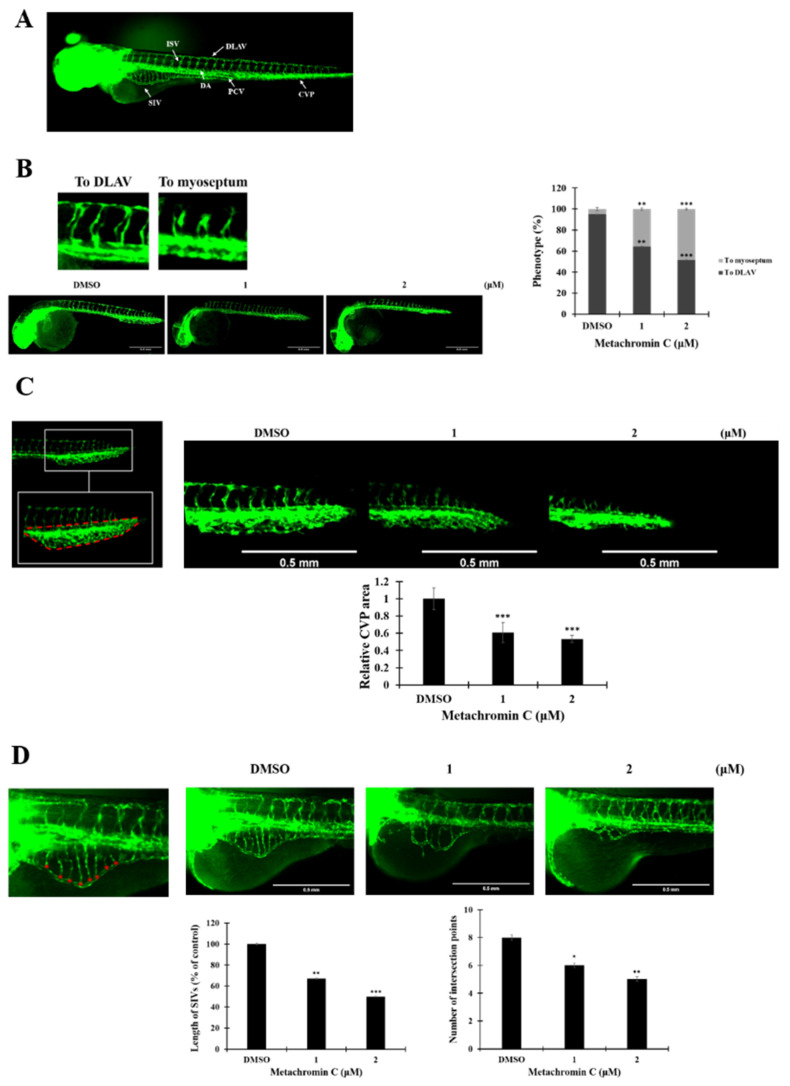
** Metachromin C inhibited the angiogenesis in zebrafish embryos.** Transgenic zebrafish Tg (fli1a: EGFP) expresses green fluorescent protein in endothelial cells. Zebrafish embryos 15 h after fertilization were treated with different concentrations of Metachromin C (1 and 2 μM) for 15 h, with DMSO as a control group, and observed using a dissecting fluorescence microscope (scale bar = 0.5 mm). (B) Schematic illustrating the blood vessel arrangement in zebrafish. Vascular standards showing intact (To DLAV) and defective (To myoseptum) intersegmental vessels (ISVs) in zebrafish. Development of zebrafish ISVs under the influence of different concentrations of Metachromin C at 30 h post-fertilization (hpf). Statistical graph showing the number of intact (To DLAV) and defective (To myoseptum) ISVs vessels in zebrafish. (C) The position of the caudal vein plexus (CVP) in zebrafish is shown, and the red dotted line represents the calculated domain of the CVP area. Development of zebrafish CVP under different concentrations of Metachromin C at 30 hpf. Statistical graph showing the relative area of the zebrafish CVP. (D) Quantitative standards for subintestinal vessels (SIVs) in zebrafish are shown, with red asterisks representing branch point locations of vascular connections of SIVs. At 3 days post-fertilization (dpf), the development of zebrafish SIVs was evaluated following exposure to different concentrations of Metachromin C. Statistical graph showing the number of branch points of SIVs in zebrafish and quantification of vessel length of SIVs in zebrafish using Image J software, the statistical graph showing the length of SIVs. Data were shown with mean ± SD (n=10). * p < 0.05, ** p < 0.01, *** p < 0.001.

**Figure 7:**
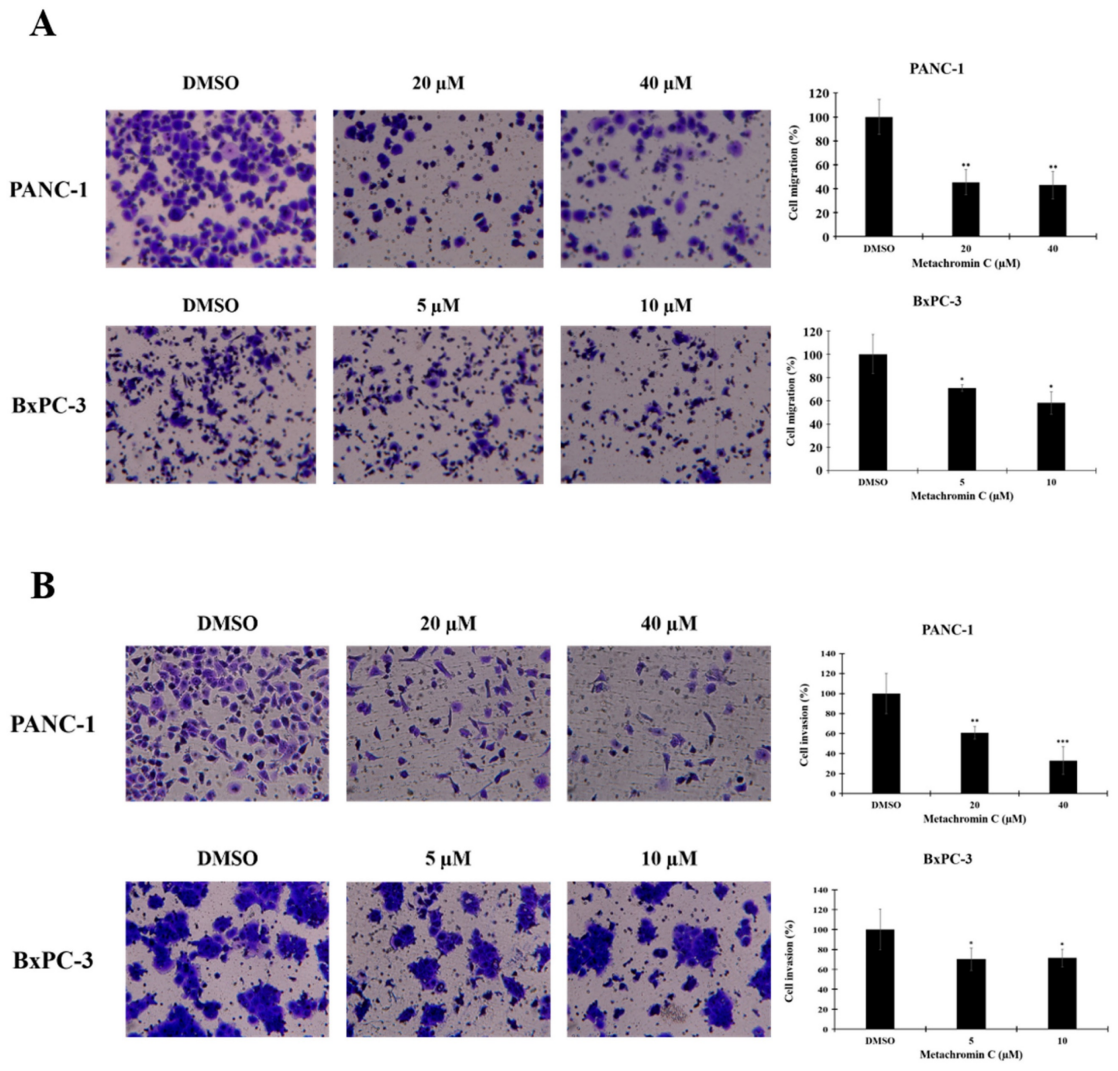
** The anti-metastatic potential of Metachromin C by inhibiting the migration and invasion ability of pancreatic cancer cells.** Transwell-migration assay of PANC-1 and BxPC-3 was conducted to investigate cell migration after indicated concentrations of Metachromin C treatment for 8 h. The migrated cells were stained with crystal violet and counted by ImageJ. Cell migration rate was normalized with the DMSO control group. (B) Cell invasion was detected by Matrigel transwell-invasion assay. PANC-1 and BxPC-3 cells were treated with Metachromin C at indicated concentrations for 24 h. The invaded cells were stained by crystal violet and counted by ImageJ. Cell invasion rate was normalized with the DMSO control group. (C) Data were shown with mean ± SD (n=3). * p < 0.05, ** p < 0.01, *** p < 0.001.

Angiogenesis contributes to cancer metastasis, and pancreatic cancer is highly aggressive with strong metastatic capacity. The transwell assay was used to study cell migration and invasion after Metachromin C treatment. PANC-1 (20 and 40 μM) and BxPC-3 (5 and 10 μM) cells were treated, and significant inhibition of migration (Fig. 5 A, and B) and invasion (Fig. 7 A, and B) was observed. These results suggest that Metachromin C can inhibit the metastatic potential of pancreatic cancer cells.

## Discussion

**Figure 8:**
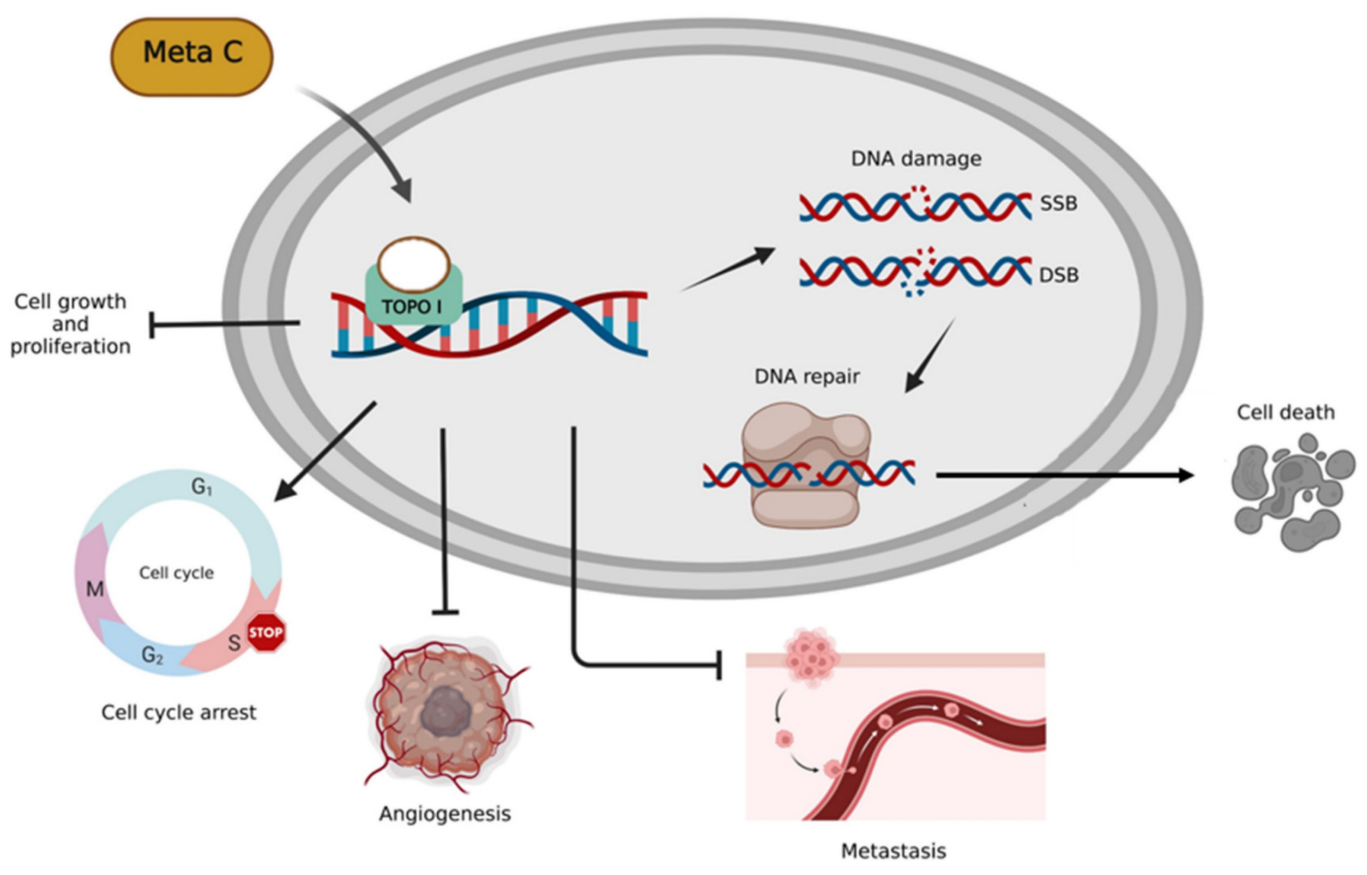
Schematic diagram of molecular mechanisms underlying the inhibitory effect of Metachromin C on pancreatic cancer and angiogenesis.

Based on our previous research, Metachromin C was found to be the most potent and lowest IC50 among dozens of natural compounds against pancreatic cancer cell lines. Metachromin C is a natural product derived from the marine sponge Hippospongia metachromia. This study showed that Metachromin C could inhibit the proliferation of pancreatic cancer cells, and its anti-cancer mechanism was to induce DNA single-strand fragmentation by inhibiting TOPO I activity, which in turn led to cell cycle arrest in the S phase. In addition, under the long-term effect of Metachromin C, the single-strand break changes to a more severe double-strand break, and then activates the DNA repair reaction, which eventually causes cell death. Similarly, Metachromin C can inhibit the migration and invasion of pancreatic cancer cells. Finally, in the angiogenesis model, Metachromin C inhibits the proliferation, migration, and tube-forming ability of HUVECs, and blocks the growth of blood vessels in zebrafish (Fig. 8). These results suggest that Metachromin C may have potential applications as a TOPO I inhibitor in cancer therapy, as well as anti-angiogenic effects.

Natural products are chemicals produced by living organisms that have pharmacological or biological activity and play an important role in the treatment of human diseases. To date, about 80% of approved cancer chemotherapy drugs are derived from natural compounds 42. However, 70% of the Earth's surface is covered by water, the vast majority of which is oceans, providing a diverse biodiversity. Sponges are the richest source of natural marine compounds of all marine organisms, accounting for 30% of all natural marine products found to date 43. Sponge products have a high chemical diversity, containing alkaloids, terpenoids, peptides, polyketones, steroids, macrolides, and other compounds, and have a variety of biological activities such as antibacterial, anticancer, antifungal, anti-HIV, and anti-inflammatory 44. In this study, Metachromin C, as a marine natural product, showed anti-cancer activity against pancreatic cancer cells and did not have much effect on normal pancreatic cells, and inhibited the newly formed blood vessels in the anti-angiogenesis effect, without affecting the pre-existing blood vessels, indicating that Metachromin C can reduce the occurrence of side effects and has the potential to be used as a precursor to anti-cancer chemotherapy drugs.

Metachromin C causes pancreatic cancer cell damage and cell cycle arrest by inhibiting the activity of TOPO I, but its anti-angiogenesis effect is not clear. In this study, we demonstrated that Metachromin C can inhibit the vascular growth of zebrafish ISVs, CVPs, and SIVs in vivo, and also can inhibit angiogenesis *in vitro*, inhibiting the proliferation, migration, and tube formation of HUVEC cells. Among a variety of pro-angiogenesis factors, VEGF has been recognized as a key factor involved in tumor angiogenesis, which is mainly secreted by tumor cells, has high specificity for endothelial cells, and plays an important regulatory role in tumor angiogenesis by binding to its receptor VEGFR2 on endothelial cells, so VEGF/VEGFR2 is a target of anti-angiogenic therapy 45. Tumor cells induce a large number of HIF-1α manifestations in response to hypoxia, which in turn activates the activation of the VEGF/VEGFR2 pathway and promotes the occurrence of angiogenesis 46. However, continuous anti-angiogenesis therapy increases tumor hypoxia and leads to increased performance of HIF-1α, which in turn promotes the production of pro-angiogenesis genes and causes tumor resistance to anti-angiogenesis therapy 47. According to the literature, Magnolol inhibits hypoxia-induced HIF-α expression and VEGF secretion by competing with VEGF for binding to VEGFR2, resulting in attenuation of kinase activity in its downstream pathway 48. However, the mechanism of Metachromin C on hypoxia-induced HIF-1α expression and VEGF/VEGFR2 signaling pathway against angiogenesis still needs to be further explored.

Rapidly proliferating cancer cells require frequent DNA replication and transcription, which depend on TOPO I and II to unwind supercoiled DNA, resulting in higher enzyme expression 49. In contrast, normal cells only require TOPO during certain cell cycle phases, leading to lower expression 50. In pancreatic cancer, over half of the cases show TOPO I overexpression 51. Metachromin C selectively inhibits TOPO in cancer cells, reducing effects on normal cells and potentially lowering treatment side effects.

DNA damage occurs during the normal metabolism of cells or is caused by environmental factors and is divided into two types: endogenous damage and exogenous damage. When DNA is damaged, cells initiate specific DNA damage responses (DDRs), including base excision repair (BER), nucleotide excision repair (NER), homologous recombination (HR), non-homologous end ligation (NHEJ) and mismatch repair (MMR), among others, send signals to repair damaged DNA and prevent irreversible damage caused by accumulated DNA damage. Activation of the DNA repair response causes various repair proteins (ATM, ATR, ChK1/2, BRCA1, WEE1, P53, p21, Cyclin B, CDC25C, and CDK1) to aggregate at the site of DNA damage for repair and arrest cell cycle progression until the damage is restored, activating the mechanism of cell death if the damage cannot be repaired 52. The laboratory results found that in pancreatic cancer cells, Metachromin C can inhibit cell growth, arrest the cell cycle in the S phase, and induce DNA damage to initiate DNA repair pathways, activating a series of DNA repair-related proteins (including ATM, ATR, P53, BRCA1, ChK1 and ChK2), which means that cancer cells try to repair damaged DNA, but it may not be enough to repair completely, and the damage cannot be recovered, which may cause cell death. In the treatment of cancer, such as chemotherapy and radiation therapy, cancer cells are induced to die by causing DNA damage. However, damaged cancer cells can initiate DNA repair pathways to resist chemotherapy drugs during treatment. Therefore, the combination of DNA repair pathway inhibitors and chemotherapeutic agents may improve the efficacy of chemotherapeutic agents on cancer cells 53, 54. Poly ADP-ribose polymerase (PARP) is a DNA repair enzyme that plays a key role in the BER, HR, and NHEJ repair pathways. When PARP activity is inhibited, the BER function cannot perform repair, resulting in the transformation of the irreparable single-strand break into a double-strand break due to the collision of replication forks, and the continuous accumulation of double-strand breaks cannot be accurately repaired by the HR pathway (for tumors with BRCA gene defects, HR repair-related proteins cannot be manufactured), resulting in cell death. For example, Olaparib, an FDA-approved PARP inhibitor for the adjuvant treatment of breast cancer patients with BRCA mutations who have received chemotherapy, has been found to have 8.8 and 7.1 percent higher rates of non-invasive disease survival and three-year distant recurrence-free survival compared with placebo in adjuvant treatment with olaparib, which may reduce recurrence and metastasis in breast cancer patients 55. BRCA mutations have been identified as risk factors for breast and ovarian cancer, and germline mutations occur in approximately 10 to 20 percent of patients with pancreatic cancer, with BRCA mutations being the most common 56. The FDA has approved olaparib as maintenance therapy for patients with pancreatic cancer with metastatic and BRCA mutations, and clinical trials have found that adjuvant olaparib is associated with 3.6 months higher progression-free survival than placebo 57. In DNA damage, double-strand breaks are the main fatal damage leading to cell death, and DNA-dependent protein kinase (DNA-PK) can participate in the repair of double-strand breaks through the NHEJ repair pathway. According to the literature, the activity of DNA-PK plays a role in resistance to radiotherapy and chemotherapy. DNA-PK inhibitors, including NU7026 58, NU7441 59, IC87361 60, and M3814 61, have been developed to inhibit the repair pathway of the double-strand break and increase the sensitivity of cancer cells to radiation therapy and chemotherapy. According to the literature, the combination of the DNA-PK inhibitor M3814 with type 2 TOPO inhibitors, including doxorubicin, etoposide, and pegylated liposomal doxorubicin, has enhanced the efficacy of type 2 TOPO inhibitors in mouse xenograft ovarian cancer models 61. Previous laboratory results have found that Metachromin C can inhibit the growth of pancreatic cancer cells and cause DNA damage to initiate DNA repair pathways, which may allow pancreatic cancer cells to repair damaged DNA, leading to the possibility that pancreatic cancer cells may become resistant to Metachromin C. Therefore, if Metachromin C is combined with DNA repair pathway inhibitors, it may make pancreatic cancer cells with damaged DNA unable to repair, resulting in more fatal damage, and ultimately cause pancreatic cancer cells to die, assisting Metachromin C in inhibiting the growth of pancreatic cancer cells.

According to the literature, the emergence of resistance to TOPO I inhibitors is often accompanied by an increase in the performance of TOPO II. Since both TOPO I and TOPO II are good targets for anti-cancer, inhibition of both may increase the activity of the compound to inhibit TOPO, causing permanent DNA damage and cell death 62. Therefore, dual inhibitors of TOPO I and TOPO II have significant therapeutic advantages over single TOPO inhibitors. In 1993, Riou *et al.* proposed that Intoplicine can inhibit both TOPO I and TOPO II, which can help improve anticancer activity against a variety of cancers 63. Wang *et al.* and He *et al.* synthesized the Ruthenium complex, which has been shown to inhibit the activity of TOPO I and TOPO II, and inhibit the growth of cervical cancer cells and liver cancer cells 64, 65. Oxocrebanine is a dual TOPO I/II inhibitor that inhibits the growth of breast cancer cells by inducing DNA damage, autophagy, and mitotic arrest 66. In this study, Metachromin C was simulated by molecular docking, and Metachromin C was shown to inhibit the activity of TOPO I by DNA relaxation assay. Therefore, further verification is needed to determine whether Metachromin C can have the effect of TOPO I/II dual inhibitors.

## Supplementary Material

Supplementary figures.

## Figures and Tables

**Table 1:** Effect of Metachromin C on zebrafish embryonic mortality.

Treatment duration	Average Survival Rate of Embryos (%)					
	DMSO	Metachromin C				
		1 µM	2 µM	2.5 µM	5 µM	10 µM
30 hpf	100	100	100	30	0	0
3 hpf	80	80	80	0	0	0
6 hpf	80	80	80	0	0	0

## References

[1] Hu C, Li M (2020). In advanced pancreatic cancer: The value and significance of interventional therapy. Journal of Interventional Medicine.

[2] Bardeesy N, DePinho RA (2002). Pancreatic cancer biology and genetics. Nature Reviews Cancer.

[3] Zhao Z, Liu W (2020). Pancreatic Cancer: A Review of Risk Factors, Diagnosis, and Treatment. Technology in Cancer Research & Treatment.

[4] Chiaravalli M, Reni M, O'Reilly EM (2017). Pancreatic ductal adenocarcinoma: State-of-the-art 2017 and new therapeutic strategies. Cancer Treatment Reviews.

[5] Dauer P, Nomura A, Saluja A, Banerjee S (2017). Microenvironment in Determining Chemo-resistance in Pancreatic Cancer: Neighborhood Matters. Pancreatology.

[6] Conroy T, Desseigne F, Ychou M, Bouché O, Guimbaud R, Bécouarn Y (2011). FOLFIRINOX versus gemcitabine for metastatic pancreatic cancer. The New England Journal of Medicine.

[7] Conroy T, Hammel P, Hebbar M, Ben Abdelghani M, Wei AC, Raoul JL (2018). FOLFIRINOX or Gemcitabine as Adjuvant Therapy for Pancreatic Cancer. The New England Journal of Medicine.

[8] Robert M, Jarlier M, Gourgou S, Desseigne F, Ychou M, Bouché O, Juzyna B, Conroy T, Bennouna J (2017). Retrospective Analysis of CA19-9 Decrease in Patients with Metastatic Pancreatic Carcinoma Treated with FOLFIRINOX or Gemcitabine in a Randomized Phase III Study (ACCORD11/PRODIGE4). Oncology.

[9] Chung S, Vail P, Witkiewicz AK, Knudsen ES (2019). Coordinately targeting cell cycle checkpoint functions in integrated models of pancreatic cancer. Clinical Cancer Research.

[10] Gupta N, Huang TT, Horibata S, Lee JM (2022). Cell cycle checkpoints and beyond: Exploiting the ATR/CHK1/WEE1 pathway for the treatment of PARP inhibitor-resistant cancer. Pharmacological Research.

[11] Bai J, Li Y, Zhang G (2017). Cell cycle regulation and anticancer drug discovery. Cancer Biology & Medicine.

[12] Watanabe K, Seki N (2024). Biology and Development of DNA-Targeted Drugs, Focusing on Synthetic Lethality, DNA Repair, and Epigenetic Modifications for Cancer: A Review. International Journal of Molecular Sciences.

[13] Matthews HK, Bertoli C, de Bruin RAM (2022). Cell cycle control in cancer. Nature Reviews Molecular Cell Biology.

[14] Groelly FJ, Fawkes M, Dagg RA, Blackford AN, Tarsounas M (2023). Targeting DNA damage response pathways in cancer. Nature Reviews Cancer.

[15] Bush NG, Evans-Roberts K, Maxwell A (2015). DNA Topoisomerases. EcoSal Plus.

[16] Clere N, Faure S, Helesbeux JJ, Duval O, Andriantsitohaina R (2011). Paradoxical effects of ethoxidine, a topoisomerase I inhibitor, in the cellular processes leading to angiogenesis on endothelial cells. Carcinogenesis.

[17] Pommier Y, Nussenzweig A, Takeda S, Austin C (2022). Human topoisomerases and their roles in genome stability and organization. Nature Reviews Molecular Cell Biology.

[18] Chen SH, Chan NL, Hsieh TS (2013). New mechanistic and functional insights into DNA topoisomerases. Annual Review of Biochemistry.

[19] Lawal B, Kuo YC, Sumitra MR, Wu ATH, Huang HS (2021). In Vivo Pharmacokinetic and Anticancer Studies of HH-N25, a Selective Inhibitor of Topoisomerase I, and Hormonal Signaling for Treating Breast Cancer. Journal of Inflammation Research.

[20] Delgado JL, Hsieh CM, Chan NL, Hiasa H (2018). Topoisomerases as Anticancer Targets. Biochemical Journal.

[21] Xu X, Liu F, Zhang S, Jia J, Li Z, Guo X (2013). Indenoisoquinoline derivatives as topoisomerase I inhibitors that suppress angiogenesis by affecting the HIF signaling pathway. Biomedicine & Pharmacotherapy.

[22] Thomas A, Pommier Y (2019). Targeting Topoisomerase I in the Era of Precision Medicine. Clinical Cancer Research.

[23] Hsiang YH, Hertzberg R, Hecht S, Liu LF (1985). Camptothecin induces protein-linked DNA breaks via mammalian DNA topoisomerase I. Journal of Biological Chemistry.

[24] Bui BP, Nguyen PL, Lee K, Cho J (2022). Hypoxia-Inducible Factor-1: A Novel Therapeutic Target for the Management of Cancer, Drug Resistance, and Cancer-Related Pain. Cancers.

[25] Masoud GN, Li W (2015). HIF-1α pathway: role, regulation and intervention for cancer therapy. Acta Pharmaceutica Sinica B.

[26] Puppo M, Battaglia F, Ottaviano C, Delfino S, Ribatti D, Varesio L (2008). Topotecan inhibits vascular endothelial growth factor production and angiogenic activity induced by hypoxia in human neuroblastoma by targeting hypoxia-inducible factor-1alpha and -2alpha. Molecular Cancer Therapeutics.

[27] Folkman J (1971). Tumor angiogenesis: therapeutic implications. The New England Journal of Medicine.

[28] Katayama Y, Uchino J, Chihara Y, Tamiya N, Kaneko Y, Yamada T (2019). Tumor Neovascularization and Developments in Therapeutics. Cancers.

[29] Liu ZL, Chen HH, Zheng LL, Sun LP, Shi L (2023). Angiogenic signaling pathways and anti-angiogenic therapy for cancer. Signal Transduction and Targeted Therapy.

[30] Lugano R, Ramachandran M, Dimberg A (2020). Tumor angiogenesis: causes, consequences, challenges and opportunities. Cellular and Molecular Life Sciences.

[31] Muz B, de la Puente P, Azab F, Azab AK (2015). The role of hypoxia in cancer progression, angiogenesis, metastasis, and resistance to therapy. Hypoxia (Auckland, NZ).

[32] Zuazo-Gaztelu I, Casanovas O (2018). Unraveling the Role of Angiogenesis in Cancer Ecosystems. Frontiers in Oncology.

[33] Vavourakis V, Wijeratne PA, Shipley R, Loizidou M, Stylianopoulos T, Hawkes DJ (2017). A Validated Multiscale In-Silico Model for Mechano-sensitive Tumour Angiogenesis and Growth. PLOS Computational Biology.

[34] Summers J, Cohen MH, Keegan P, Pazdur R (2010). FDA Drug Approval Summary: Bevacizumab plus Interferon for Advanced Renal Cell Carcinoma. The Oncologist.

[35] Yang T, Xiao H, Liu X, Wang Z, Zhang Q, Wei N (2021). Vascular Normalization: A New Window Opened for Cancer Therapies. Frontiers in Oncology.

[36] Newman DJ, Cragg GM (2012). Natural products as sources of new drugs over the 30 years from 1981 to 2010. Journal of Natural Products.

[37] Han BN, Hong LL, Gu BB, Sun YT, Wang J, Liu JT (2019). Natural Products from Sponges. Symbiotic Microbiomes of Coral Reefs Sponges and Corals.

[38] Lu WY, Li HJ, Li QY, Wu YC (2021). Application of marine natural products in drug research. Bioorganic & Medicinal Chemistry.

[39] Dyshlovoy SA, Honecker F (2019). Marine Compounds and Cancer: The First Two Decades of XXI Century. Marine Drugs.

[40] Kobayashi J, Murayama T, Ohizumi Y, Ohta T, Nozoe S, Sasaki T (1989). Metachromin C, a new cytotoxic sesquiterpenoid from the Okinawan marine sponge Hippospongia metachromia. Journal of Natural Products.

[41] Shen YC, Chen CY, Kuo YH (2001). New sesquiterpene hydroquinones from a Taiwanese marine sponge, Hippospongia metachromia. Journal of Natural Products.

[42] Newman DJ, Cragg GM (2016). Natural Products as Sources of New Drugs from 1981 to 2014. Journal of Natural Products.

[43] Mehbub MF, Lei J, Franco C, Zhang W (2014). Marine sponge derived natural products between 2001 and 2010: trends and opportunities for discovery of bioactives. Marine Drugs.

[44] Essack M, Bajic VB, Archer JAC (2011). Recently confirmed apoptosis-inducing lead compounds isolated from marine sponge of potential relevance in cancer treatment. Marine Drugs.

[45] Ferrara N, Adamis AP (2016). Ten years of anti-vascular endothelial growth factor therapy. Nature Reviews Drug Discovery.

[46] Krock BL, Skuli N, Simon MC (2011). Hypoxia-induced angiogenesis: good and evil. Genes and Cancer.

[47] Jayson GC, Kerbel R, Ellis LM, Harris AL (2016). Antiangiogenic therapy in oncology: current status and future directions. The Lancet.

[48] Chen MC, Lee CF, Huang WH, Chou TC (2013). Magnolol suppresses hypoxia-induced angiogenesis via inhibition of HIF-1α/VEGF signaling pathway in human bladder cancer cells. Biochemical Pharmacology.

[49] Liu J, Geng G, Liang G, Wang L, Luo K, Yuan J (2020). A novel topoisomerase I inhibitor DIA-001 induces DNA damage mediated cell cycle arrest and apoptosis in cancer cell. Annals of Translational Medicine.

[50] Bronstein IB, Vorobyev S, Timofeev A, Jolles CJ, Alder SL, Holden JA (1996). Elevations of DNA topoisomerase I catalytic activity and immunoprotein in human malignancies. Oncology Research.

[51] Heestand GM, Schwaederle M, Gatalica Z, Arguello D, Kurzrock R (2017). Topoisomerase expression and amplification in solid tumours: Analysis of 24,262 patients. European Journal of Cancer.

[52] Sirbu BM, Cortez D (2013). DNA Damage Response: Three Levels of DNA Repair Regulation. Cold Spring Harbor Perspectives in Biology.

[53] Li LY, Guan YD, Chen XS, Yang JM, Cheng Y (2021). DNA Repair Pathways in Cancer Therapy and Resistance. Frontiers in Pharmacology.

[54] Carlsen L, El-Deiry WS (2022). Anti-cancer immune responses to DNA damage response inhibitors: Molecular mechanisms and progress toward clinical translation. Frontiers in Oncology.

[55] Tutt ANJ, Garber JE, Kaufman B, Viale G, Fumagalli D, Rastogi P (2021). Adjuvant Olaparib for Patients with BRCA1- or BRCA2-Mutated Breast Cancer. The New England Journal of Medicine.

[56] Petersen GM (2016). Familial pancreatic cancer. Seminars in Oncology.

[57] Golan T, Hammel P Reni M, Van Cutsem E Macarulla T, Hall MJ et al (2019). Maintenance Olaparib for Germline BRCA-Mutated Metastatic Pancreatic Cancer. The New England Journal of Medicine.

[58] Dolman ME, van der Ploeg I, Koster J, Bate-Eya LT, Versteeg R, Caron HN (2015). DNA-Dependent Protein Kinase As Molecular Target for Radiosensitization of Neuroblastoma Cells. PLOS ONE.

[59] Yang C, Wang Q, Liu X, Cheng X, Jiang X, Zhang Y (2016). NU7441 Enhances the Radiosensitivity of Liver Cancer Cells. Cellular Physiology and Biochemistry.

[60] Yang H, Yao F, Marti TM, Schmid RA, Peng RW (2020). Beyond DNA Repair: DNA-PKcs in Tumor Metastasis, Metabolism and Immunity. Cancers.

[61] Wise HC, Iyer GV, Moore K, Temkin SM, Gordon S, Aghajanian C (2019). Activity of M3814, an Oral DNA-PK Inhibitor, In Combination with Topoisomerase II Inhibitors in Ovarian Cancer Models. Scientific Reports.

[62] Salerno S, Da Settimo F, Taliani S, Simorini F, La Motta C, Fornaciari G (2010). Recent advances in the development of dual topoisomerase I and II inhibitors as anticancer drugs. Current Medicinal Chemistry.

[63] Riou JF, Fossé P, Nguyen CH, Larsen AK, Bissery MC, Grondard L (1993). Intoplicine (RP 60475) and its derivatives, a new class of antitumor agents inhibiting both topoisomerase I and II activities. Cancer Research.

[64] Wang YC, Qian C, Peng ZL, Hou XJ, Wang LL, Chao H (2014). Dual topoisomerase I and II poisoning by chiral Ru(II) complexes containing 2-thiophenylimidazo[4,5-f][1,10]phenanthroline derivatives. Journal of Inorganic Biochemistry.

[65] He X, Jin L, Tan L (2015). DNA-binding, topoisomerases I and II inhibition and in vitro cytotoxicity of ruthenium(II) polypyridyl complexes: [Ru(dppz)2L](2+) (L=dppz-11-CO2Me and Dppz). Spectrochimica Acta Part A: Molecular and Biomolecular Spectroscopy.

[66] Yu L, Han S, Lang L, Song H, Zhang C, Dong L (2021). Oxocrebanine: A Novel Dual Topoisomerase Inhibitor, Suppressed the Proliferation of Breast Cancer Cells MCF-7 by Inducing DNA Damage and Mitotic Arrest. Phytomedicine.

