# Supplementary material for: Metachromin C, a marine-derived natural compound, shows potential in antitumor activity
Source: Int J Med Sci. 2024 Oct 7;21(13):2578–94. doi: 10.7150/ijms.101037 (PMC11492879; doi:10.7150/ijms.101037)
Supplement: Supplementary file 1 — Supplementary figures. [file ijmsv21p2578s1.pdf]

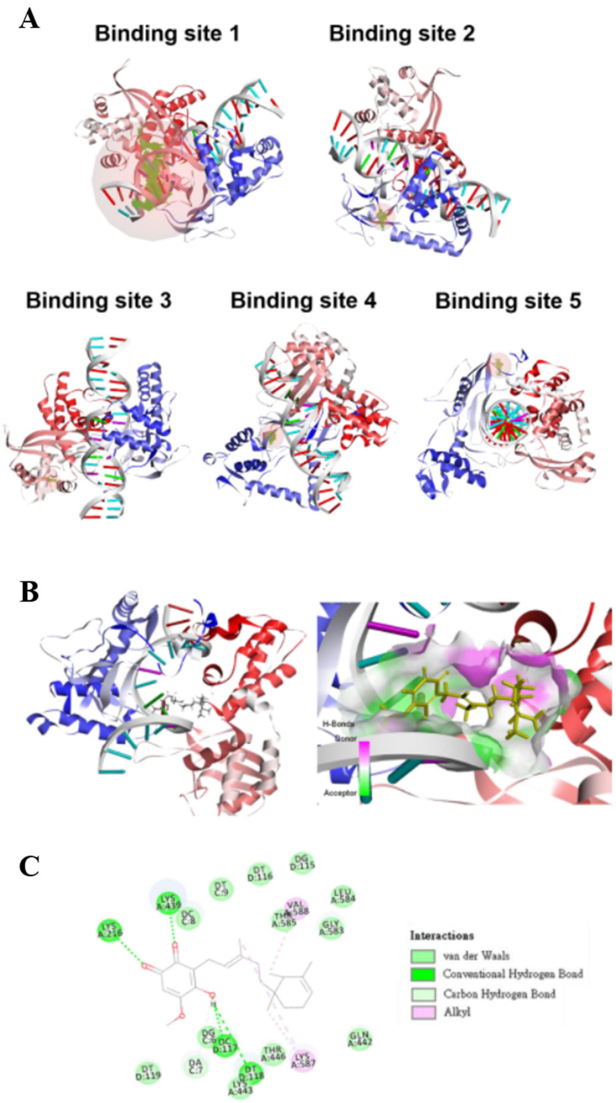

Figure S1. Metachromin C could dock into the DNA binding site of TOPO1.

(A) Three possible docking sites of Metachromin C, cited as 1-5, were found in the interface of the TOPO 1-DNA complex. Site 1 was the best choice for docking simulation. (B) Detailed interaction of Metachromin C at site 1. A LigandFit module was used to find the docking sites of Metachromin C at the interface of the TOPO1-DNA complex. (C) The interaction between Metachromin C and TOPO 1, the

hydrogen bonds formed at Lys216 and Lys439 of TOPO1 were shown.

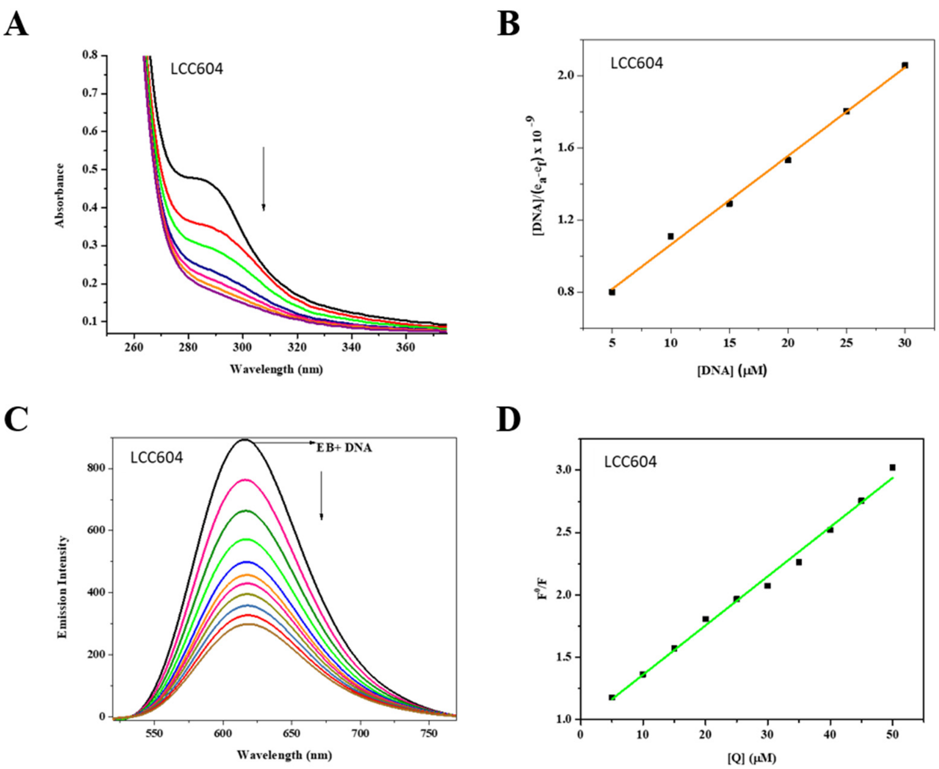

Figure S2. The binding mode of Metachromin C to CT DNA is through intercalation.

(A) Absorption spectra of Metachromin C in Tris-HCl buffer upon addition of CT-DNA. [Metachromin C] = 2.0 μM, [DNA] = 5–30 μM. The arrow shows that the absorption intensity decreases upon increasing the DNA concentration. (B) Plot of [DNA]/ (εa − εf) versus [DNA] for the titration of the title compound with CT-DNA. (C) Fluorescence quenching curve of EB bound to DNA for Metachromins C. [DNA] = 5 μM, [EB] = 5 μM and [METACHROMIN C] = 0–50 μM. (D) Stern–Volmer graph representing the fluorescence titration of Metachromin C with CT-DNA.
